# Rubipodanin A, the First Natural *N*-Desmonomethyl Rubiaceae-Type Cyclopeptide from *Rubia podantha*, Indicating an Important Role of the *N* ^9^-Methyl Group in the Conformation and Bioactivity

**DOI:** 10.1371/journal.pone.0144950

**Published:** 2015-12-22

**Authors:** Zhe Wang, Si-Meng Zhao, Li-Mei Zhao, Xiao-Qiang Chen, Guang-Zhi Zeng, Ning-Hua Tan

**Affiliations:** 1 State Key Laboratory of Phytochemistry and Plant Resources in West China, Kunming Institute of Botany, Chinese Academy of Sciences, Kunming, 650201, PR China; 2 Department of Natural Medicinal Chemistry & State Key Laboratory of Natural Medicines, China Pharmaceutical University, Nanjing, 210009, PR China; 3 University of Chinese Academy of Sciences, Beijing, 100049, PR China; Nanyang Technological University, SINGAPORE

## Abstract

One new cyclic hexapeptide named rubipodanin A (**1**), which is the first identified natural *N*-desmonomethyl Rubiaceae-type cyclopeptide, together with six known Rubiaceae-type cyclopeptides (**2**–**7**) were obtained using the TLC cyclopeptide protosite detection method with ninhydrin from the roots and rhizomes of *Rubia podantha*. The cyclopeptide structures were elucidated by extensive spectroscopic analysis, including 1D-NMR, 2D-NMR, IR, UV and MS. The solution conformation and biological activities of **1** and RA-V (**4**) were evaluated, and the results demonstrated that the *N* ^9^-methyl group plays a vital role in the maintenance of the conformation and bioactivity.

## Introduction

Rubiaceae-type cyclopeptides (RAs) are homodicyclohexapeptides mainly composed of one *α*-D-alanine, one *α*-L-alanine, three *N*-methyl-*α*-L-tyrosines, and one other proteinogenic *α*-L-amino acid. The most unusual feature is a 14-membered ring formed by a phenolic oxygen linkage between two adjacent tyrosines with a *cis* peptide bond, and the 14-membered ring is fused to a 18-membered cylic hexapeptide ring [1,2]. In 1977, bouvardin and deoxybouvardin (RA-V), the first two RAs, were isolated from *Bouvardin ternifolia* (Rubiaceae) with potential antitumor activities [3]. Subsequently, an additional 34 RAs have been isolated from *Rubia codifolia*, *R*. *yunnanensis* and *R*. *akane*, and more than 200 analogues have been synthesized [1,4–8]. Studies on antitumor mechanisms of RAs have indicated that RA-VII suppresses protein synthesis through interaction with eukaryotic 80S ribosomes [9]. Furthermore, RA-V and RA-XII inhibit the production of NO and inducible nitric oxide synthase (iNOS) [10]. Moreover, RA-VII has the ability to change the conformational structure of F-actin to induce G_2_ arrest [11].

For the unique bicyclic structure and significant antitumor activities *in vitro* and *in vivo* [1,2,12], RAs have recently attracted our interest. We have now isolated 28 RAs and synthesized several RA analogues, including 11 novel natural RAs consisting of two new skeleton RAs and one new *O*-seco-RA from *R*. *yunnanensis* and *R*. *schumanniana*, respectively [13–17]. We have also evaluated their cytotoxic activities and performed 2D- and 3D-QSAR studies on 54 RAs [18]. Our studies showed that RA-V exhibits anti-inflammatory activity by inhibiting NO production and NF-κB activation induced by TNF-α [13]. Furthermore, RA-V significantly suppresses angiogenesis by down-regulating ERK1/2 phosphorylation in HUVEC and HMEC-1 endothelial cells [19]. In addition, RA-V kills human breast cancer cells by inducing mitochondria-mediated apoptosis and inhibits cell adhesion and invasion via the PI3K/AKT and NF-κB signaling pathways [20,21]. Importantly, the potential roles of RAs in cancer therapy have been highlighted [22].

Some investigations have shown that *N*-methylated amino acids of cyclopeptides play an important role in the maintenance of their conformation and bioactivity. Our previous NMR studies have indicated that both natural and synthetic RAs have two or four conformers [13–18], which prompted us to obtain the natural *N*-desmethyl RAs and investigate the functional role of amino acid *N*-methylation in conformation and bioactivity. In this study, we selected another *Rubia* plant, *Rubia podantha* Diels, which has been used as a substitute for the traditional Chinese medicine *R*. *codifolia*. The roots and rhizomes have been used to treat tuberculosis, menoxenia, rheumatism, contusion, hematemesis, anemia and lipoma for a long time in China. To the best of our knowledge, however, there is no literature on the chemical and antitumor constitutes of this plant. To expand the distribution of plant resources containing RAs and explore the functional role of *N*-methylated amino acids in natural RAs in the maintenance of the conformation and bioactivity, we structurally and pharmacologically investigated interesting RAs using the TLC cyclopeptide protosite detection method with ninhydrin [23] from *R*. *podantha*. Fortunately, a new cyclic hexapeptide, rubipodanin A (**1**), which is the first identified natural *N*-desmonomethyl RA, together with six known RAs (**2–7**) were obtained. Here, the isolation and structural elucidation of **1**–**7** as well as cytotoxic and NF-κB signaling pathway activities of **1** and **4** are described.

## Materials and Methods

### General experimental procedures

Optical rotations were obtained on a Jasco P-1020 polarimeter. IR spectra were measured by a Tensor 27 spectrometer using KBr pellets. UV spectra were performed using a Shimadzu UV-2401A spectrophotometer. 1D-NMR and 2D-NMR spectra were recorded on a Bruker AVANCE III-600 and Ascend ^™^ 800 spectrometer at 298K. Chemical shifts (*δ*) were expressed in parts per million (ppm) with reference to the solvent signals. Mass spectra were obtained on a Waters XEVO-TQD spectrometer or an Agilent G6230 TOF Mass spectrometer. Analytical or semi-preparative HPLC was performed on an Agilent 1100 with a Zorbax Eclipse-C18 (4.6 mm × 150 mm; 9.4 mm × 250 mm; 5 μm).

Column chromatography was performed with silica gel (100–200 mesh and 200–300 mesh, Qingdao Yu-Ming-Yuan Chemical Co. Ltd., Qingdao, China), Sephadex LH-20 (Pharmacia Fine Chemical Co., Uppsala, Sweden) or Lichroprep RP-18 gel (40–63 μm, Merck, Darmstadt, Germany). Fractions were monitored by TLC (GF254, Qingdao Yu-Ming-Yuan Chemical Co. Ltd., Qingdao, China), and orange spots were visualized on the plate by spraying with 2% ninhydrin reagent after hydrolyzed in a drying incubator (110°C) for 30 min by HCl [23].

### Plant material

The roots and rhizomes of *R*. *podantha* were collected in November of 2013 from Mount Chang Chong, Kunming, China. The locations/activities to collect *R*. *podantha* did not require specific permission because this studied plant is common in the field in Yunnan, China and is not cultivated in privately owned or protected areas. In addition, the filed studies did not involve endangered and protected species. The material was identified by Professor Ning-Hua Tan of Kunming Institute of Botany, Chinese Academy of Sciences. A voucher specimen (KUN0359279) was deposited in the Herbarium of Kunming Institute of Botany.

### Extraction and isolation

The air-dried and powdered roots and rhizomes of *R*. *podantha* (13 kg) were extracted three times with methanol (3 × 15 L) under reflux. After removal of the solvent under vacuum, the methanol extract (1.63 kg) was subjected to silica gel column chromatography (CC) eluted with petroleum ether-CHCl_3_ (1:0, 1:1, 0:1) and CHCl_3_-MeOH (95:5, 9:1, 8:2, 7:3, 0:1) to afford eight fractions. Fractions containing cyclopeptides (150 g, CHCl_3_-MeOH, 9:1) were rechromatographed on a silica gel CC eluted with a CHCl_3_-MeOH gradient system (70:1–8:2) to yield six fractions (Fr.1-Fr.6).

Fr.2 (40 g) was applied to silica gel CC using petroleum ether-acetone (3:1–0:1) to yield five subfractions (Fr.2-1 to Fr.2-5). Fr.2-3 (17 g) was separated using Sephadex LH-20 CC (CHCl_3_-MeOH, 1:1) and then purified over repeated silica gel CC eluting with petroleum ether-acetone (3:2) to afford RA-V (**4**) (878 mg).

Fr.3 (37 g) was chromatographed on a silica gel CC using a CHCl_3_-MeOH system (100:1–8:2) to yield six subfractions (Fr.3-1 to Fr.3-6). Fr.3-3 (21 g) was subjected to RP-18 CC, eluting with MeOH-H_2_O (20–90%) to afford five subfractions (Fr.3-3-1 to Fr.3-3-5). Fr.3-3-2 (9 g) were separated by Sephadex LH-20 CC (CHCl_3_-MeOH, 1:1) and purified over repeated silica gel CC eluting with petroleum ether-acetone (2:1) to afford RA-VII (**5**) (658 mg). Fr.3-3-3 (3 g) was also passed through Sephadex LH-20 CC (CHCl_3_-MeOH, 1:1) and then silica gel CC (petroleum ether–acetone, 3:2) to yield four subfrations (Fr.3-3-3-1 to Fr.3-3-3-4). Fr.3-3-3-2 (32 mg) was further purified by semi-preparative HPLC (30% CH_3_CN) to yield rubipodanin A (**1**) (9 mg). Fr.3-3-3-3 (43 mg) was also further purified by semi-preparative HPLC (35% CH_3_CN) to yield RA-I (**2**) (7 mg) and RA-III (**3**) (8 mg).

Fr.4 (10 g) was subjected to an RP-18 CC, eluting with MeOH-H_2_O (20–90%) to afford four subfractions. Fr.4-4-2 (4 g) were separated by Sephadex LH-20 CC (CHCl_3_-MeOH, 1:1) and then further purified by semi-preparative HPLC (30% CH_3_CN) to yield Allo-RA-V (**7**) (4 mg).

Fr.5 (32 g) was submitted to silica gel CC eluting with gradient CHCl_3_-MeOH (50:1–8:2) to yield five subfractions (Fr.5-1 to Fr.5-5). Fr.5-3 (13 g) was further purified by repeated silica gel CC (CHCl_3_-MeOH, 20:1–8:2) followed by Sephdex LH-20 CC (CHCl_3_-MeOH, 1:1), and it was further separated by repeated RP-18 CC (MeOH-H_2_O, 30–70%), which led to the isolation of RA-XII (**6**) (927 mg).

Rubipodanin A (**1**): white amorphous powder; [α]18.5 D -215.5 (*c* 0.06, CHCl_3_); UV (MeOH) λ_max_ (log ε) 203 (4.84), 222 (4.58), 278 (3.73) nm; IR (KBr) ν_max_ 3428, 2957, 2925, 2871, 1656, 1636, 1514, 1500, 1459, 1411, 1377, 1288, 1246, 1212, 1182, 1160, 1111, 1094, 1033 cm^-1^; ^1^H (800 MHz) and ^13^C (200 MHz) NMR data, see Table 1; positive ESIMS m/z 743.80 [M+H]^+^; positive HRESIMS m/z 765.3214 [M+Na]^+^, calcd for C_39_H_46_N_6_NaO_9_ 765.3224.

**Table 1:** ^1^H-NMR (800 MHz) and ^13^C-NMR (200 MHz) spectral data of rubipodanin A (1) in C_5_D_5_N (*δ* in ppm, *J* in Hz).

Residues	Position	*δ*_H_	*δ*_C_
D-Ala-1	α	5.18 (overlap; 1H)	48.3 d
	β	1.55 (d; 7.0; 3H)	21.8 q
	C = O		172.6 s
	N-H	8.90 (d; 8.0; 1H)	
L-Ala-2	α	4.91 (m; 1H)	49.0 d
	β	1.46 (d; 6.9; 3H)	17.0 q
	C = O		173.0 s
	N-H	9.80 (d; 8.5; 1H)	
L-Tyr-3	α	4.10 (m; 1H)	57.8 d
	βa	3.98 (t; 13.5; 1H)	33.9 d
	βb	3.85 (dd; 13.5; 3.7; 1H)	
	γ		132.9 s
	δ * 2	7.33 (d; 8.6; 2H)	131.1 d
	ε * 2	7.04 (d; 8.6; 2H)	114.4 d
	ζ		158.9 s
	C = O		169.9 s
	N-H	10.59 (d; 6.9; 1H)	
	OMe	3.71 (s; 3H)	55.3 q
L-Ala-4	α	5.19 (overlap; 1H)	46.8 d
	β	1.40 (d; 6.7; 3H)	19.5 q
	C = O		172.0 s
	N-H	7.81 (d; 8.1; 1H)	
L-Tyr-5	α	5.77 (dd; 11.4; 3.2; 1H)	54.6 d
	βa	3.65 (t; 11.4; 1H)	36.7 t
	βb	2.60 (dd; 11.4; 3.2; 1H)	
	γ		136.2 s
	δa	7.24 (m; 1H)	131.0 d
	δb	7.43 (dd; 8.3; 2.2; 1H)	133.5 d
	εa	6.94 (dd; 8.3; 2.2; 1H)	126.5 d
	εb	6.89 (dd; 8.3; 2.2; 1H)	124.6 d
	ζ		158.9 s
	C = O		169.9 s
	NMe	3.00 (s; 3H)	30.4 q
L-Tyr-6	α	5.01 (m; 1H)	58.0 d
	βa	3.34 (dd; 17.8; 12.0; 1H)	36.5 t
	βb	3.54 (dd; 17.8; 3.0; 1H)	
	γ		128.1 s
	δa	6.76 (d; 8.2; 1H)	122.2 d
	δb	4.60 (s; 1H)	115.1 d
	εa	7.22 (overlap; 1H)	117.9 d
	εb		152.7 s
	ζ		145.5 s
	C = O		171.5 s
	NMe	3.03 (s; 3H)	29.8 q

### Cell culture

HeLa, A549, SGC7901 and HEK293T cell lines were purchased from the American Type Culture Collection (ATCC, USA) and were cultured in Dulbecco’s modified Eagle’s medium (Invitrogen, USA) supplemented with 10% (v/v) heat-inactivated fetal bovine serum (FBS) (Life technologies, USA) and 1% (v/v) penicillin-streptomycin (Invitrogen, USA). The cells were maintained at 37°C in a humidified incubator atmosphere of 5% CO_2_/95% air (v/v).

### Cytotoxicity assay

Cytotoxicity was tested by the Sulforhodamine B (SRB) method (Sigma, USA). The assays were performed as described previously [24].

### Cell transfection and luciferase assay

HEK293T cells were seeded in 24-well plates and transiently transfected with 5×κB-luciferase and pTK-Renilla reporters using Lipofectamine 2000 (Invitrogen, USA) for 18 h. The cells were then incubated with different concentrations of compounds for the indicated time, and subsequently stimulated with 10 ng/ml TNF-α for 2 h. The luciferase activity of the cell lysate was analyzed by the Dual Luciferase Reported Assay System (Promega, USA). Luciferase reporter assays were performed as described previously [25].

### Western blot analysis

Cells were lysed using RIPA buffer containing 50 mM Tris-HCl (pH 7.4), 150 mM NaCl, 1 mM EDTA, 1% Triton X-100, 0.5% deoxycholate, 0.1% SDS, protease inhibitors and phosphatase inhibitors. After being disrupted on ice for 30 min, the cell lysates were centrifuged at 12,000 rpm for 15 min and boiled with SDS sample buffer at 95°C for 5 min. The samples were equally subjected to SDS-PAGE, electrophoresed, and transferred to PVDF membranes (Millipore, USA). After blocking with 5% nonfat milk in TBST, the membranes were incubated with the indicated antibody for 2 h or overnight at 4°C, and the membranes were then incubated with horseradish peroxidase (HRP)-conjugated secondary antibody for 1 h at room temperature. The protein bands were detected with a SuperSingal West Pico Chemiluminescence ECL kit (Pierce, USA).

### Statistical analysis

Student’s t-test was used for statistical analysis. A *p* value < 0.05 was considered to be statistically significant.

## Results and Discussion

**Fig 1:**
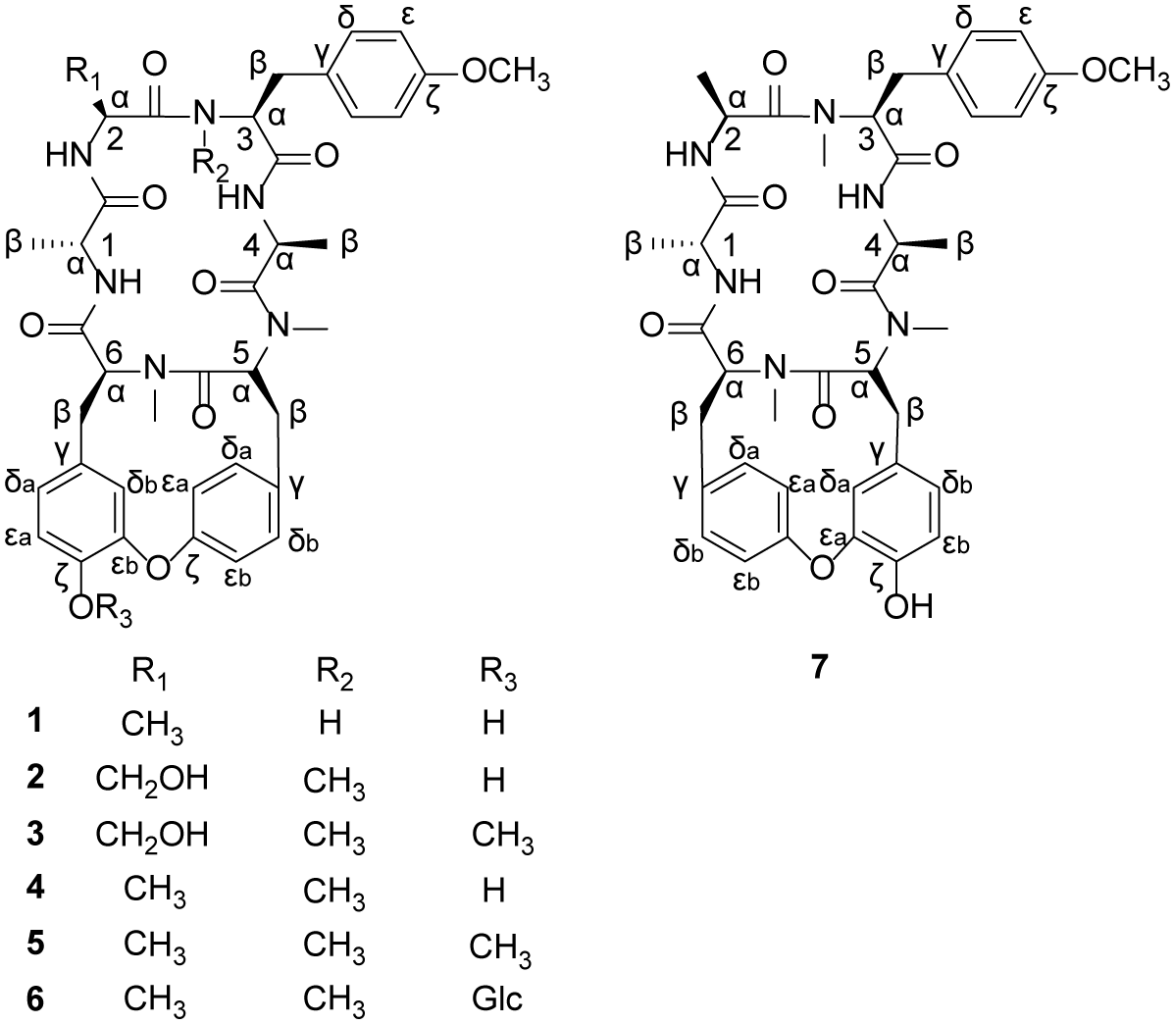
Chemical structures of 1–7.

The methanol extract of the air-dried powdered roots and rhizomes of *R*. *podantha* was subjected to fractionation using silica gel CC, which yielded the total cyclopeptide fraction guided by the TLC cyclopeptide protosite detection method with ninhydrin [23]. This fraction was repeatedly chromatographed over a series of silica gel, Sephadex LH-20, RP-18 CC, and ODS HPLC to obtain seven RAs (Fig 1), including one new natural *N*-demonomethyl RA (rubipodanin A, **1**) and six known RAs, including RA-I (**2**), RA-III (**3**), RA-V (**4**), RA-VII (**5**), RA-XII (**6**) and Allo-RA-V (**7**). The structure and stereochemistry of **1** was established by 1D-NMR, 2D-NMR, UV, IR and MS, and the structural identification of known compounds **2–7** was performed by comparison with NMR data in the literature [7,13,26–28]. Furthermore, cytotoxic activities of **1** and **4** against three tumor cell lines and inhibitory activities of **1** and **4** on the NF-κB signaling pathway were investigated. The results of the solution conformation and biological activities of **1** and **4** suggested that the *N* ^9^-methyl group plays a vital role in the maintenance of their conformation and bioactivity.

### Characterization of Rubipodanin A (1)

**Fig 2:**
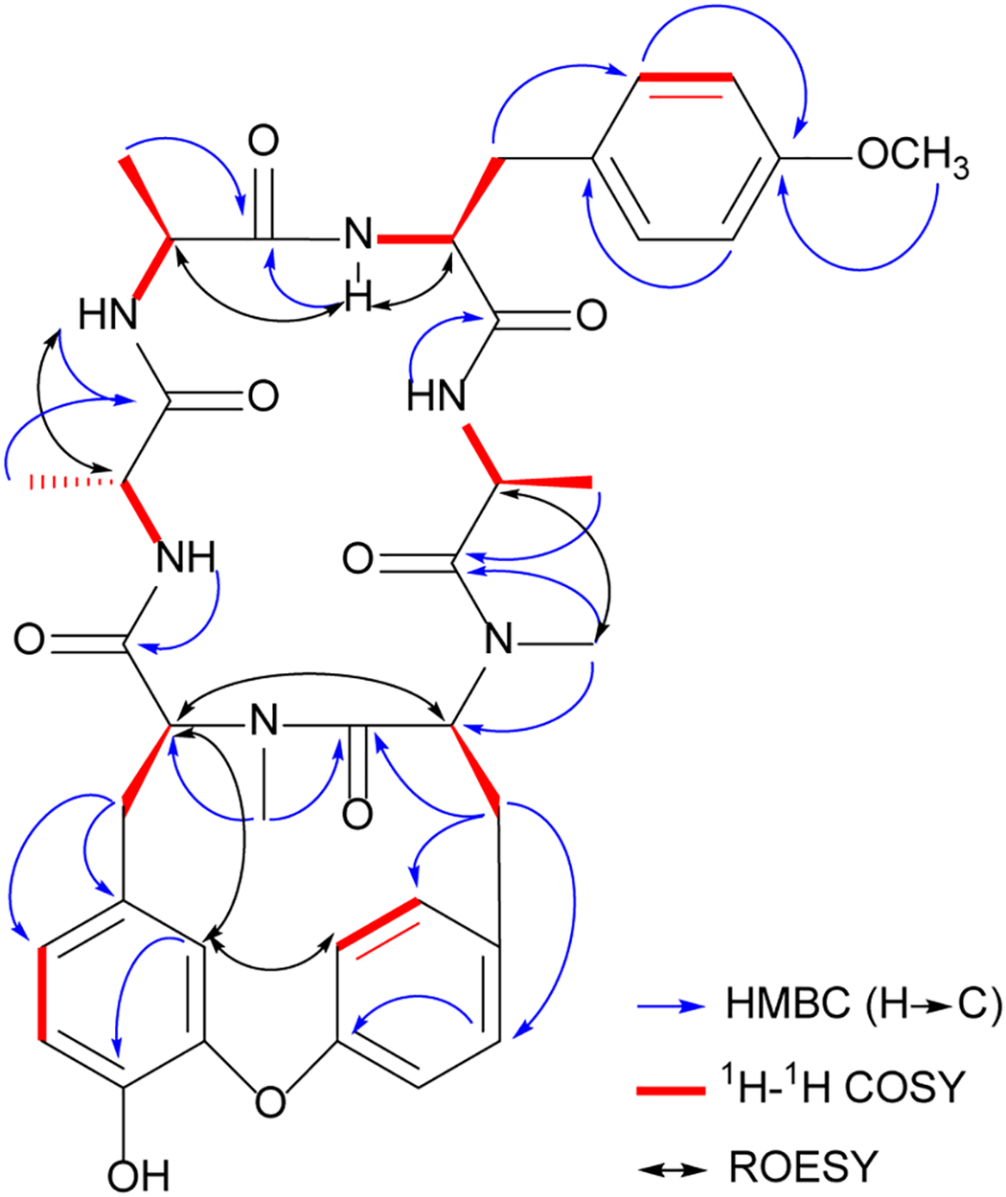
Key HMBC, ^1^H-^1^H COSY, and ROESY correlations of 1.

Rubipodanin A (**1**) was obtained as white amorphous powder, and its molecular formula was established as C_39_H_46_N_6_O_9_ by its positive HRESIMS (m/z 765.3214, [M+Na]^+^), indicating 20 degrees of unsaturation. The UV spectrum of rubipodanin A showed absorptions at 203, 222 and 278 nm, which demonstrated the existence of phenyl groups. The IR spectrum exhibited absorption bands at 3428 and 1636 cm^-1^, which is indicative of OH, NH and CO groups. The ^1^H and ^13^C NMR spectra of **1** in C_5_D_5_N (Table 1) displayed characteristics of typical RAs without any conformers, but these characteristics are common in natural RAs. Further analysis of 1D-NMR and 2D-NMR spectra data of **1** displayed the signals for the three methyls (*δ*_H_/*δ*_C_ 1.55/21.8, 1.46/17.0, 1.40/19.5), two amide N-methyls (*δ*_H_/*δ*_C_ 3.03/29.8, 3.00/30.4), one O-methyl (*δ*_H_/*δ*_C_ 3.71/55.3), three methylenes (*δ*_H_/*δ*_C_ 3.85, 3.98/33.9; 2.60, 3.65/36.7; 3.34, 3.54/36.5), six α-amino methines (*δ*_H_/*δ*_C_ 5.77/54.6, 5.19/46.8, 5.18/48.3, 5.01/58.0, 4.91/49.0, 4.10/57.8), two 1,4-disubstituted benzene rings (*δ*_C_ 158.9, 132.9 and *δ*_H_/*δ*_C_ 7.33/131.1 × 2, 7.04/114.4 × 2; *δ*_C_ 158.9, 136.2 and *δ*_H_/*δ*_C_ 7.43/133.5, 7.24/131.0, 6.94/126.5, 6.89/124.6), one 1,2,4-trisubstituted benzene ring (*δ*_C_ 152.7, 145.5, 128.1, and *δ*_H_/*δ*_C_ 7.22/117.9, 6.76/122.2, 4.60/115.1), six carbonyl signals (*δ*_C_ 173.0, 172.6, 172.0, 171.5, 169.9, 169.9) and four amide protons (*δ*_H_ 10.59, 9.80, 8.90, 7.81). An extensive comparison of 1D-NMR and 2D-NMR spectra data of **1** with those of RA-V (**4**) in C_5_D_5_N indicated that both compounds were similar, except for the third amino acid residue (Tyr^3^). Further analysis of the HMBC and ^1^H-^1^H COSY correlations confirmed the construction of the *N*-desmethyl Tyr^3^ residue (Fig 2). In the ^1^H-^1^H COSY spectrum, cross-peaks of NH-3/H-3α/H-3β suggested that the amide *N* ^9^-methyl was absent, which was also confirmed by the HMBC correlation from *δ*_H_ 10.59 (NH-3) to *δ*_c_ 173.0 (C-2-CO). In addition, the sequence of the amino acid residues in **1** was confirmed by the HMBC correlations from *δ*_H_ 9.80 (2-NH) to *δ*_c_ 172.6 (C-1-CO), from *δ*_H_ 10.59 (3-NH) to *δ*_c_ 173.0 (C-2-CO), from *δ*_H_ 7.81 (4-NH) to *δ*_c_ 169.9 (C-3-CO), from *δ*_H_ 3.00 (H-5NCH_3_) to *δ*_c_ 172.0 (C-4-CO), from *δ*_H_ 3.03 (H-6NCH_3_) to *δ*_c_ 169.9 (C-5-CO) and from *δ*_H_ 8.90 (1-NH) to *δ*_c_ 171.5 (C-6-CO). In the ROESY spectrum, NOE correlations were observed among 3-NH/H-2α, H-3α, 5-NCH_3_/H-4α, and H-5α/H-6α, thereby indicating that the peptide bonds between Ala^2^/Tyr^3^, Ala^4^/Tyr^5^ and Tyr^5^/Tyr^6^ were *trans*, *trans*, and *cis*, respectively. Because previous have studies demonstrated that the absolute configurations of Ala^1^ and Ala^4^ in RAs were generally D (*R*) and L (*S*) [13], the remaining configurations of Ala^2^, Tyr^3^, Tyr^5^ and Tyr^6^ were deduced as L (*S*), L (*S*), L (*S*) and L (*S*), respectively, by observing the following NOE correlations: H-1α/2-NH; 3-NH/H-2α, H-3α; 5-NCH_3_/H-4α; and H-5α/H-6α. Collectively, the structure of **1** was identified as shown in Fig 1, which is the first identified natural *N*-desmonomethyl RA.

**Fig 3:**
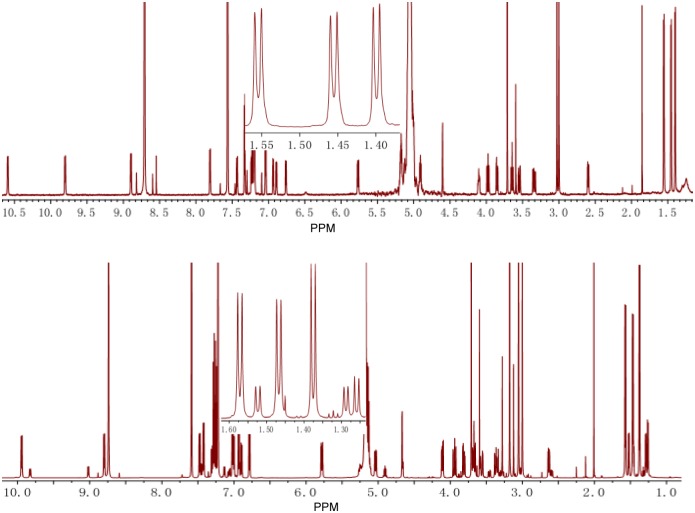
^1^H NMR spectra comparison of rubipodanin A (1, top) and RA-V (4, bottom).

The *N*-methylated amino acids of cyclopeptides play an important role for the conformation in solution, and RAs generally possess two or four conformers, including natural RAs, synthetic RAs and *O*-seco-RAs. In 1995, the DL Boger group synthesized several *N*-desmethyl derivatives of RA-VII and investigated their conformation property by ^1^H-NMR spectra, and they reported that both *N* ^9^- and *N* ^15^-methyl groups are essential for maintenance of their conformation [29–32], which prompted us to investigate the difference between **1** and **4** in NMR spectra. Consistent with previous studies [31], the ^1^H NMR spectra of **4** in C_5_D_5_N displayed the presence of two conformers in a ratio of 79:21, and **1** revealed a single solution conformation (Fig 3). The similar phenomenon occurred in ^13^C NMR spectra (Figure N in S1 File), which demonstrated that the *N* ^9^-methyl group plays a vital role in the conformational property of RAs.

### Biological Assay

Some studies have shown that natural bioactive cyclopeptides contain at least one *N*-methylated amino acid. For example, the *N*-methyl groups of cyclosporin A (CsA), a cyclic undecapeptide containing seven *N*-methylated amino acids, play an important role for maintenance of immunosuppressive activity [33]. Sansalvamide A, isolated from a marine fungus of the genus *Fusarium*, is a cyclic tetrapeptide, and the *N*-methyl derivatives of sansalvamide A exhibit better antitumor activity [34].

**Fig 4:**
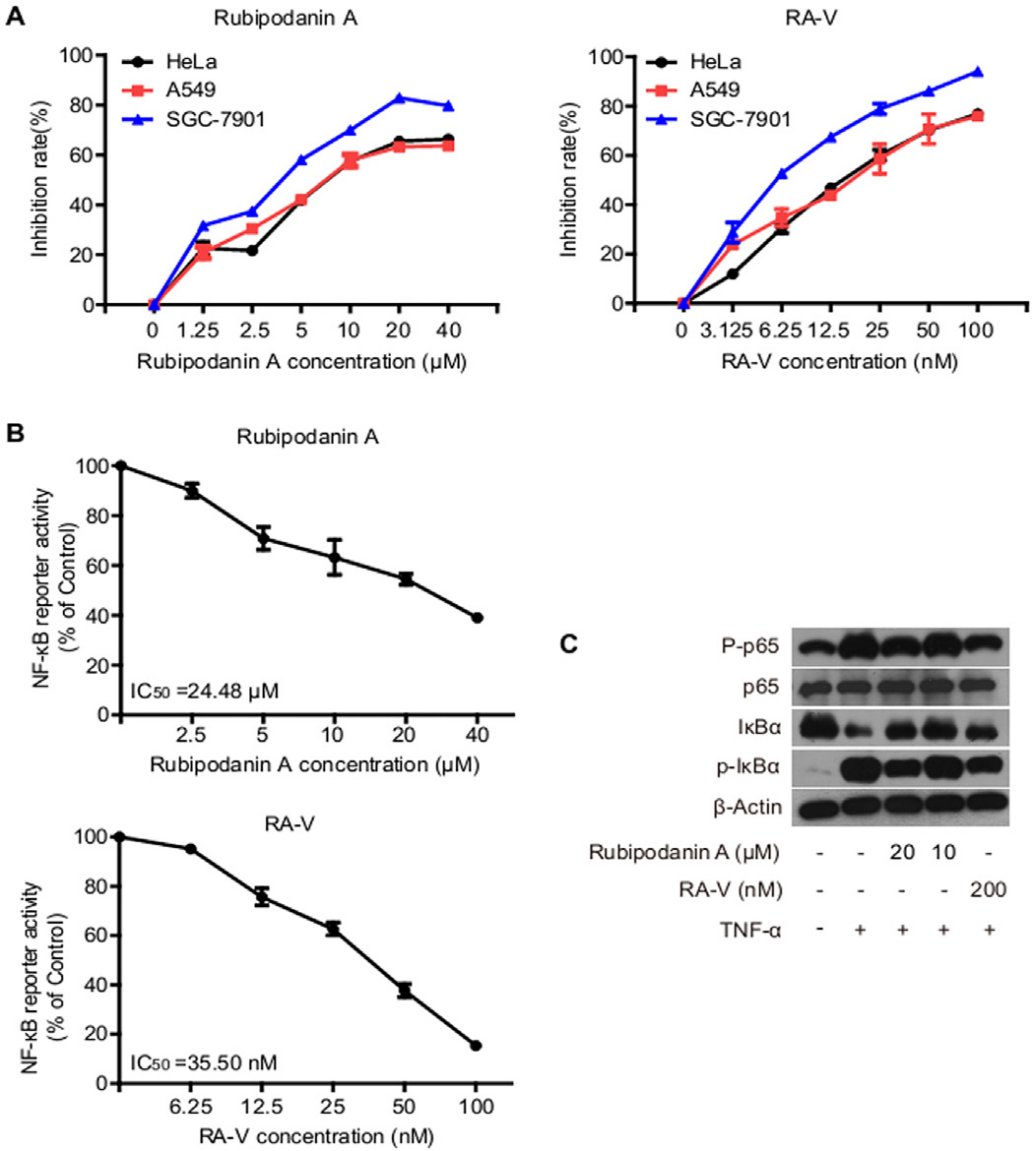
The cytotoxicity and inhibitory activity of rubipodanin A (1) and RA-V (4) against the NF-κB signaling pathway.

RAs are a type of potent antitumor agent and often contain three *N*-methylated tyrosines, which promotes researchers to explore the functional role of *N*-methyl groups on their biological activity. The DL Boger group evaluated the cytotoxicity of synthetic *N*-desmethyl RA derivatives and showed that the *N* ^15^-methyl group is essential for their biological activities [32]. In the present study, we investigated the cytotoxicity against three tumor cell lines and inhibitory activity against the tumor-associated NF-κB signaling pathway of rubipodanin A (**1**) compared to RA-V (**4**) by using the SRB assay and the Dual Luciferse Reporter Assay System, respectively. As shown in Fig 4, 1 had weak cytotoxicity against HeLa, A549 and SGC-7901 with IC_50_ values of 7.22 ± 0.76, 7.14 ± 0.81, 3.80 ± 0.17 μM, respectively, which was 420–650 times less than **4** (Table 2). We also found that **4** exhibited a potent inhibitory activity against the NF-κB pathway activation induced by TNF-α, which was approximately 700 times greater than that of **1**. In addition, we examined the effects of **1** and **4** on the expression of NF-κB-associated proteins using western blot analysis. As expected, **1** also had an inhibitory effect on TNF-α-induced IκBα phosphorylation, IκBα degradation and p65 phosphorylation only in the high concentration up to 20 μM, while **4** exhibited similar inhibitory effect at a concentration 200 nM. Taken together, these results suggested that the *N* ^9^-methyl group is essential for maintenance of biological activities of natural RAs and that down-regulation of the NF-κB pathway might account for the mechanism of the antitumor activities of RAs. Of note, previous reports have shown that the *N* ^9^-methyl group of synthetic RAs is not critical for biological activities [32], which was not consistent with our results. So more *N*-desmethyl derivatives of RAs from plants or by synthesis in future are needed to confirm this difference based on the structure-activity relationship analysis.

**Table 2:** Cytotoxicity of rubipodanin A (1) and RA-V (4) compounds.

	1	4
HeLa	7.22 ± 0.76	0.015 ± 0.0014
A549	7.14 ± 0.81	0.017 ± 0.0026
SGC-7901	3.80 ± 0.17	0.0058 ± 0.0016

(IC_50_, μM and Mean ±SD)

## Conclusion

The phytochemical studies of *Rubia podantha* were performed using the TLC cyclopeptide protosite detection method with ninhydrin, which led to the identification of one new cyclic hexapeptide (rubipodanin A, **1**) and six known ones (**2**–**7**). Importantly, **1** is the first identified natural *N*-desmonomethyl RA. Further studies revealed that **1** had only one solution conformation in NMR but two in RA-V (**4**), and it also had lower cytotoxicity and inhibitory activity against the NF-κB signaling pathway than **4**, thus indicating that the *N* ^9^-methyl group is essential for the conformation and biological activities of RAs. These findings not only expanded the distribution of plant resources containing RAs but also provided new insights into the role of the *N*-methyl group in maintaining conformational and biological properties of RAs.

## Supporting Information

S1 File Relevant spectra of Rubipodanin A (1) and RA-V (4). ^1^H NMR Spectrum of Rubipodanin A (**1**) (**Figure A**). ^13^C NMR Spectrum of Rubipodanin A (**1**) (**Figure B**). HSQC Spectrum of Rubipodanin A (**1**) (**Figure C**). COSY Spectrum of Rubipodanin A (**1**) (**Figure D**). HMBC Spectrum of Rubipodanin A (**1**) (**Figure E**). ROESY Spectrum of Rubipodanin A (**1**) (**Figure F**). ESI Mass Spectrum of Rubipodanin A (**1**) (**Figure G**). High Resolution Mass Spectrum of Rubipodanin A (**1**) (**Figure H**). UV Spectrum of Rubipodanin A (**1**) (**Figure I**). IR Spectrum of Rubipodanin A (**1**) (**Figure J**). [α]^D^ Spectrum of Rubipodanin A (**1**) (**Figure K**). ^1^H NMR Spectrum of RA-V (**4**) (**Figure L**). ^13^C NMR Spectrum of RA-V (**4**) (**Figure M**). ^13^C NMR spectra comparison of Rubipodanin A (**1,** top) and RA-V (**4,** bottom) (**Figure N**).
(DOC)
