# Supplementary material for: Rubipodanin A, the First Natural N-Desmonomethyl Rubiaceae-Type Cyclopeptide from Rubia podantha, Indicating an Important Role of the N 9-Methyl Group in the Conformation and Bioactivity
Source: PLoS One. 2015 Dec 22;10(12):e0144950. doi: 10.1371/journal.pone.0144950 (PMC4687918; doi:10.1371/journal.pone.0144950)

**Supporting Information**

**Rubipodanin A, the First Natural *N*-Desmonomethyl Rubiaceae-Type Cyclopeptide from *Rubia podantha*, Indicating an Important Role of the *N*^9^-Methyl Group in the Conformation and Bioactivity**

**Zhe Wang^1,3¶^, Si-Meng Zhao^1^**^¶^**, Li-Mei Zhao^1^, Xiao-Qiang Chen^1^, Guang-Zhi Zeng^1^*, Ning-Hua Tan^1,2^***

^1^ State Key Laboratory of Phytochemistry and Plant Resources in West China, Kunming Institute of Botany, Chinese Academy of Sciences, Kunming 650201, PR China

^2^ Department of Natural Medicinal Chemistry & State Key Laboratory of Natural Medicines, China Pharmaceutical University, Nanjing 210009, PR China

^3^ University of Chinese Academy of Sciences, Beijing 100049, PR China

Contents

|  |
| --- |
| Figure A. ^1^H NMR Spectrum of Rubipodanin A (**1**) |
| Figure B.^13^C NMR Spectrum of Rubipodanin A (**1**) |
| Figure C. HSQC Spectrum of Rubipodanin A (**1**) |
| Figure D. COSY Spectrum of Rubipodanin A (**1**) |
| Figure E. HMBC Spectrum of Rubipodanin A (**1**) |
| Figure F. ROESY Spectrum of Rubipodanin A (**1**) |
| Figure G. ESI Mass Spectrum of Rubipodanin A (**1**) |
| Figure H. High Resolution Mass Spectrum of Rubipodanin A (**1**) |
| Figure I. UV Spectrum of Rubipodanin A (**1**) |
| Figure J. IR Spectrum of Rubipodanin A (**1**) |
| Figure K. [α]^D^ Spectrum of Rubipodanin A (**1**) |
| Figure L. ^1^H NMR Spectrum of RA-V (**4**) |
| Figure M. ^13^C NMR Spectrum of RA-V (**4**) |
| Figure N. ^13^C NMR spectra comparison of Rubipodanin A (**1,** top)   and RA-V (**4,** bottom) |

Figure A. ^1^H NMR Spectrum of Rubipodanin A (**1**)
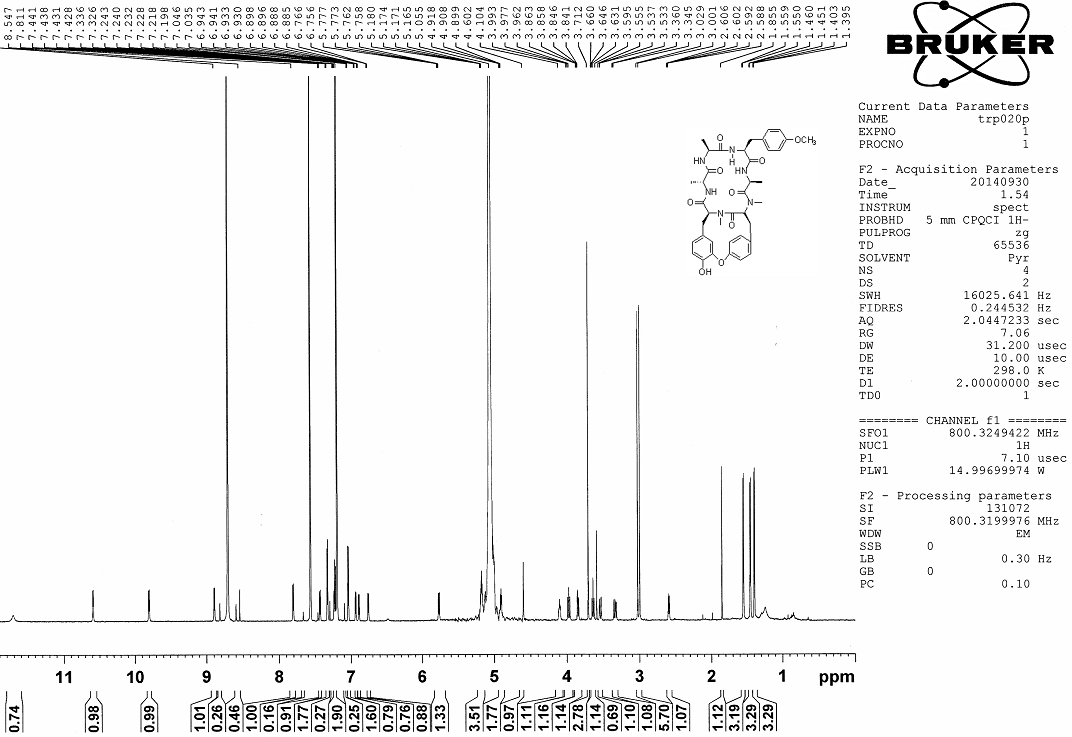


Figure B.^13^C NMR Spectrum of Rubipodanin A (**1**)
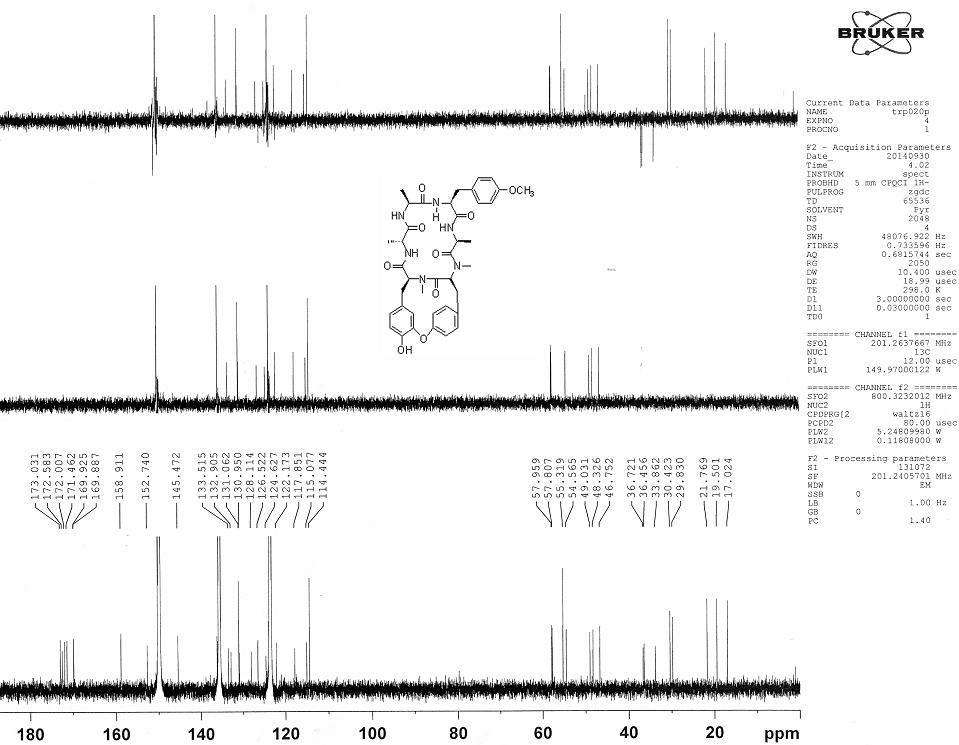


Figure C. HSQC Spectrum of Rubipodanin A (**1**)


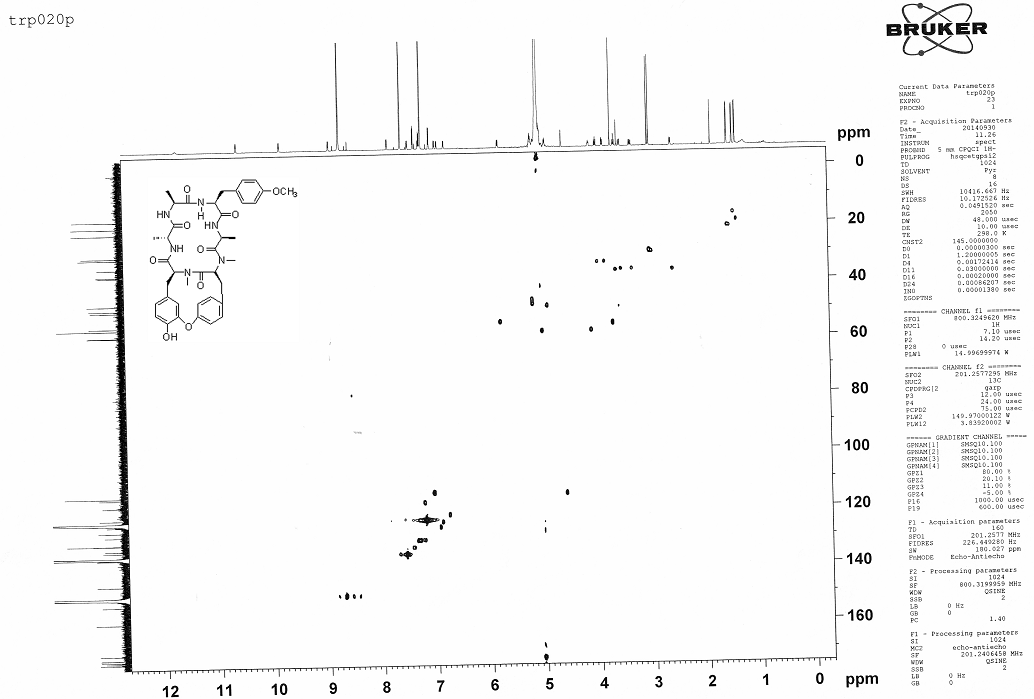


Figure D. COSY Spectrum of Rubipodanin A (**1**)


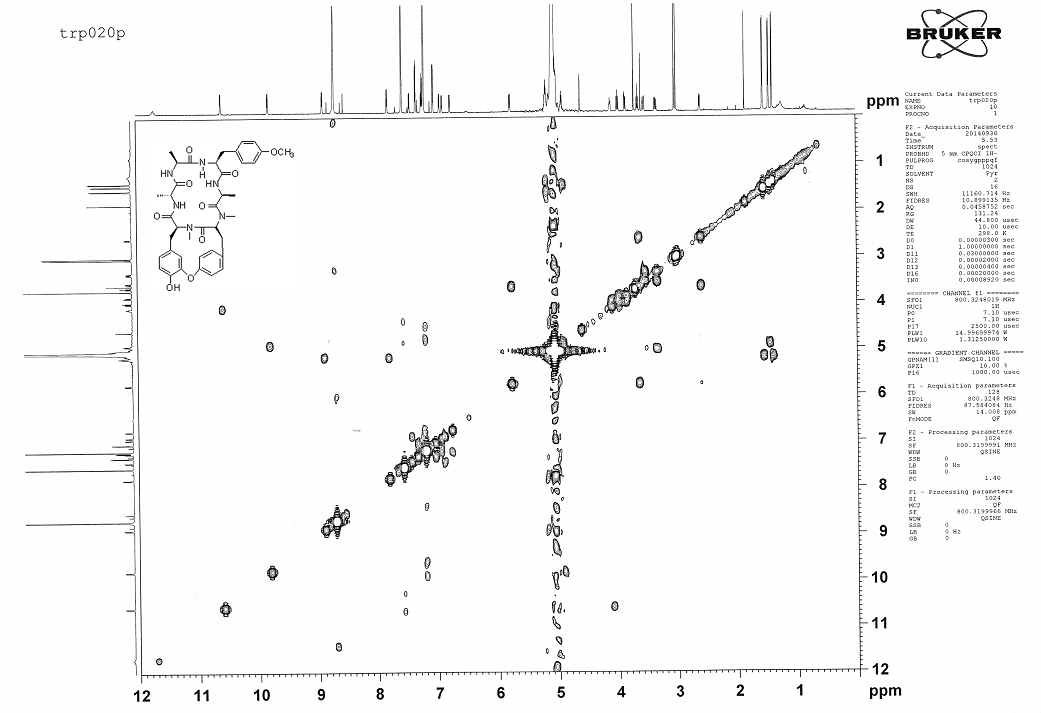


Figure E. HMBC Spectrum of Rubipodanin A (**1**)
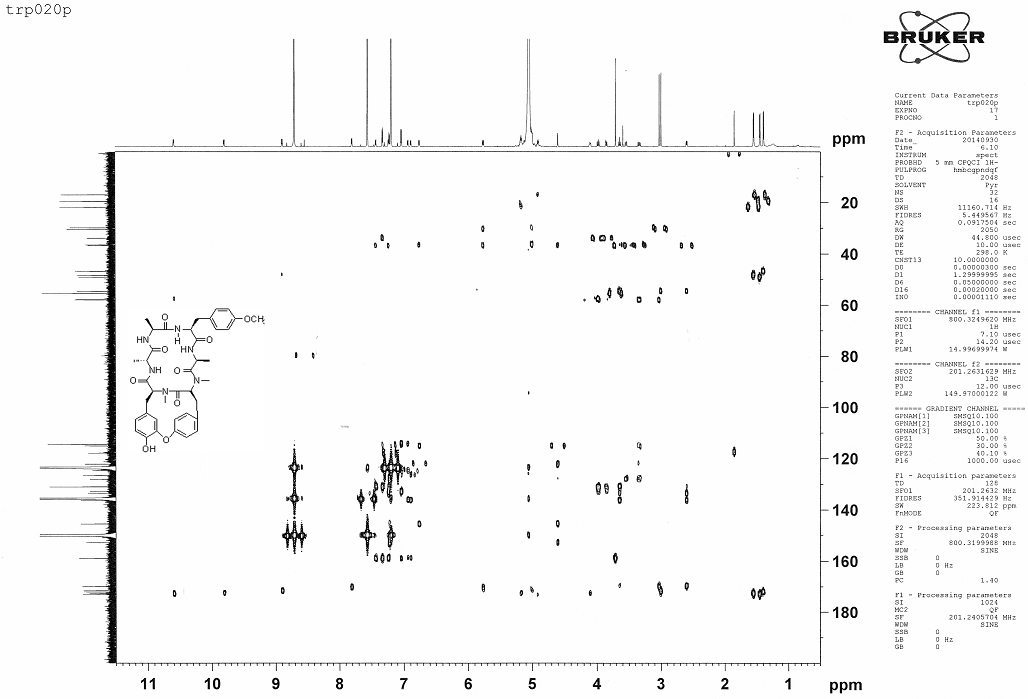


Figure F. ROESY Spectrum of Rubipodanin A (**1**)
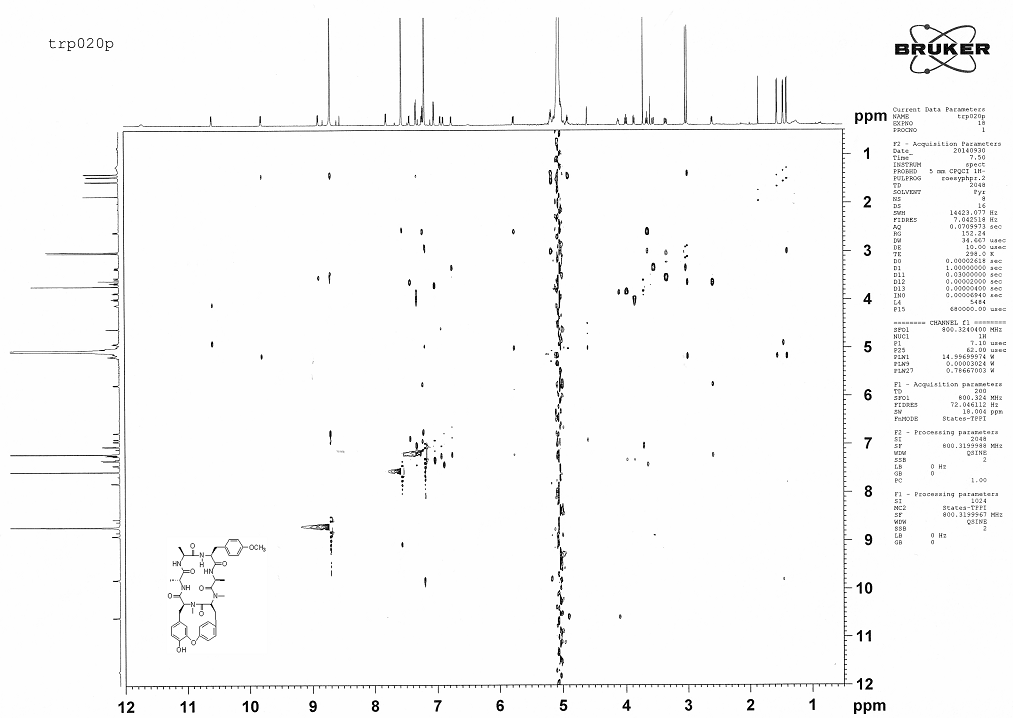


Figure G. ESI Mass Spectrum of Rubipodanin A (**1**)
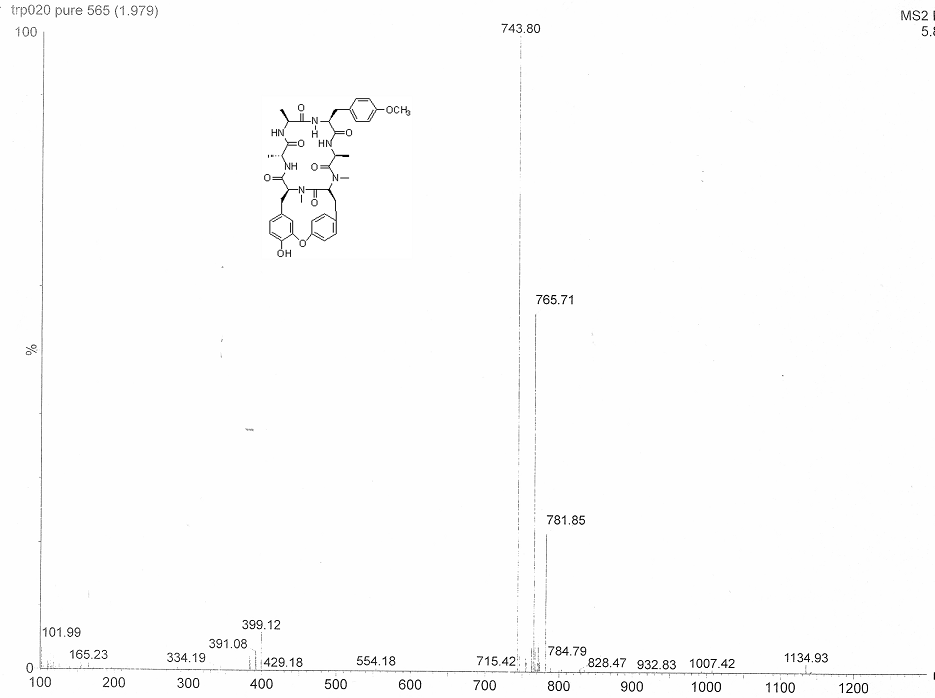


Figure H. High Resolution Mass Spectrum of Rubipodanin A (**1**)


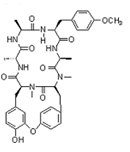

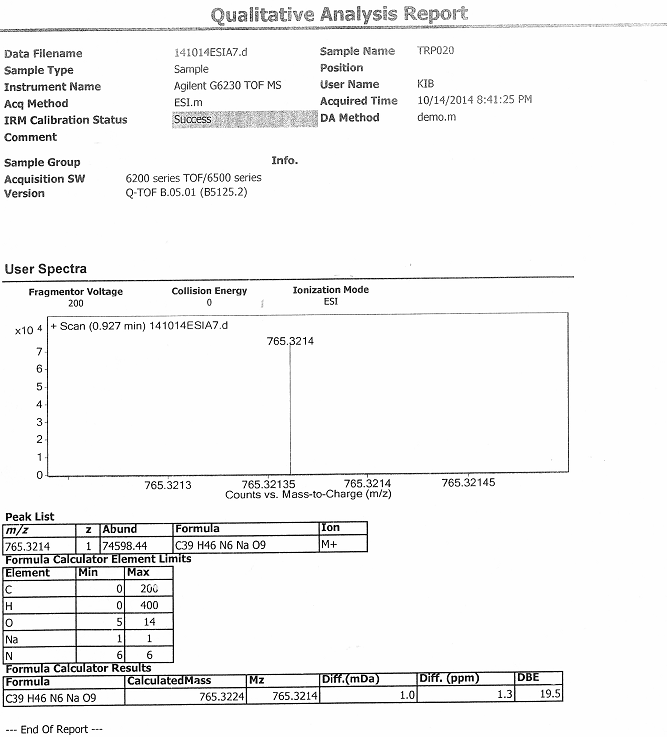


Figure I. UV Spectrum of Rubipodanin A (**1**)


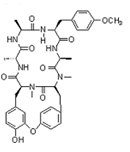

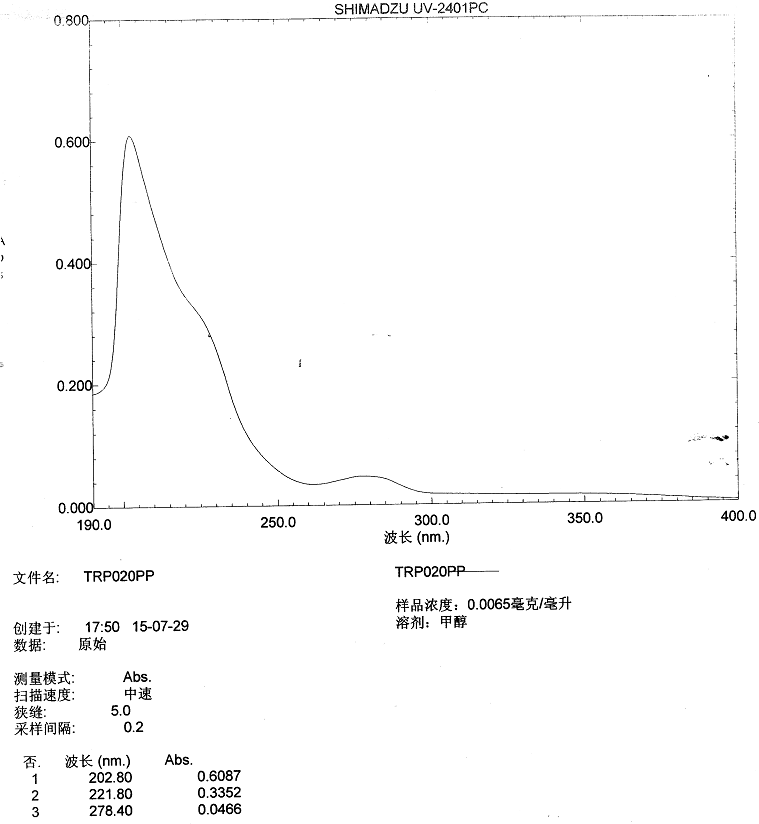


Figure J. IR Spectrum of Rubipodanin A (**1**)


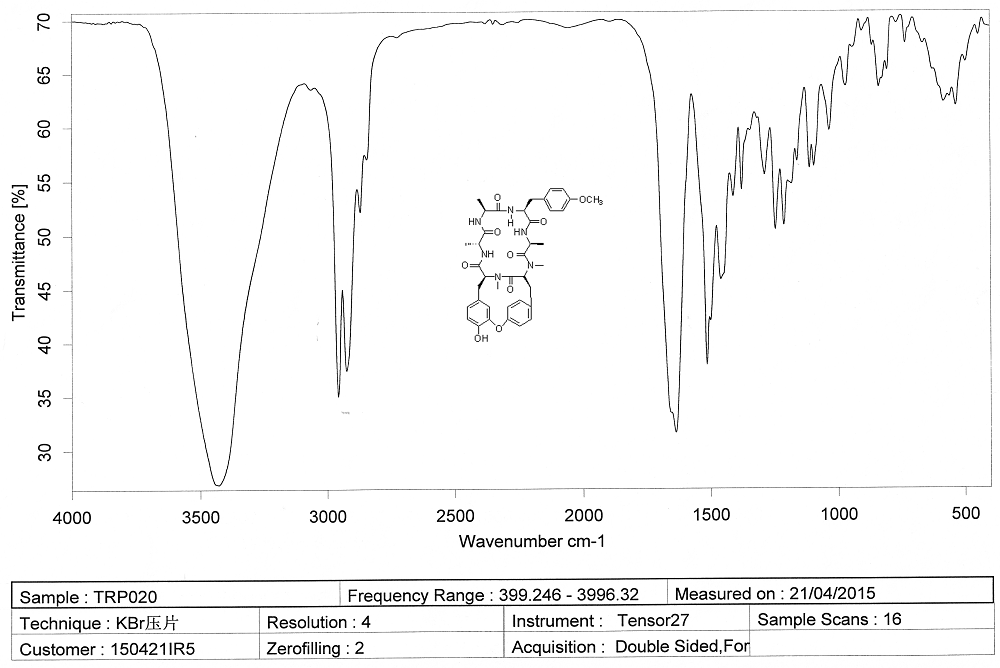


Figure K. [α]^D^ Spectrum of Rubipodanin A (**1**)


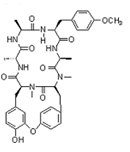

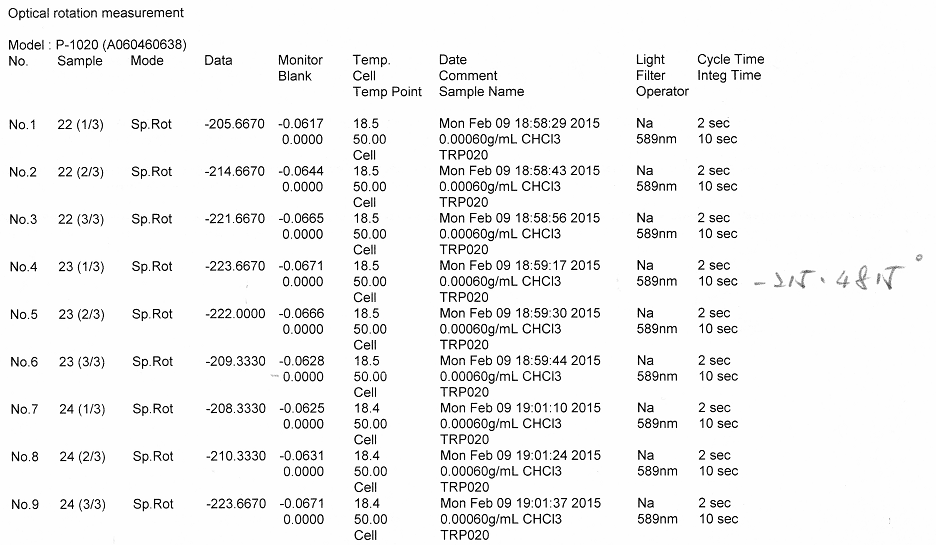


Figure L. ^1^H NMR Spectrum of RA-V (**4**)


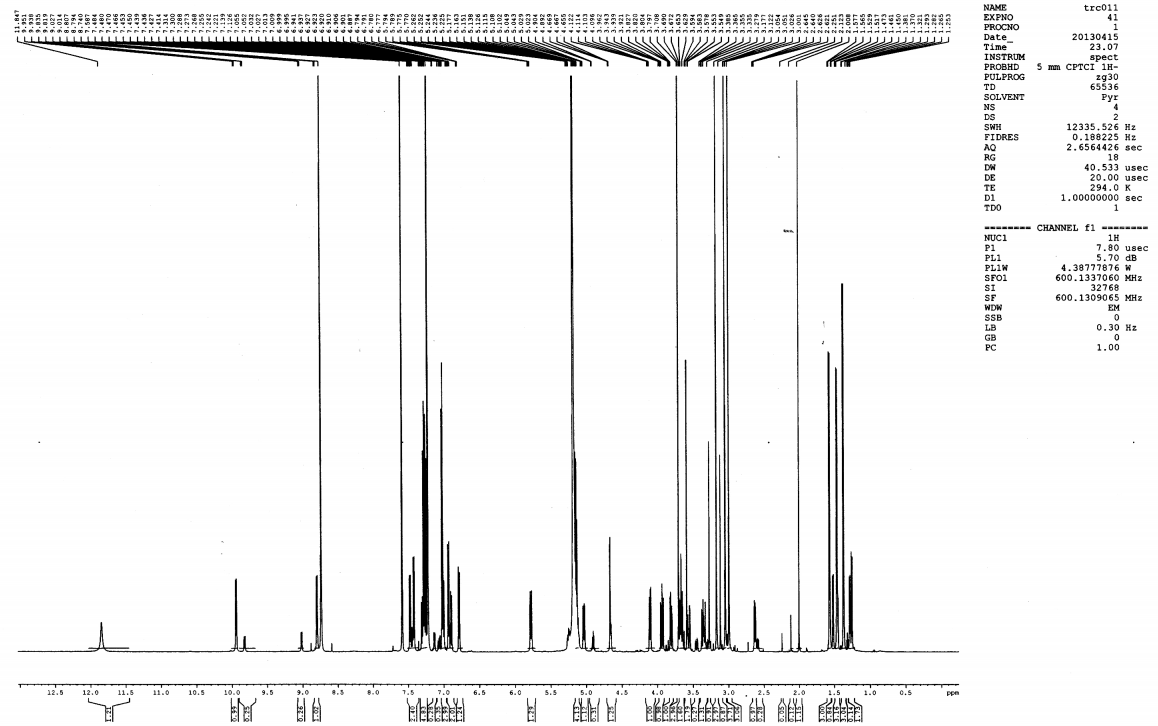


Figure M. ^13^C NMR Spectrum of RA-V (**4**)


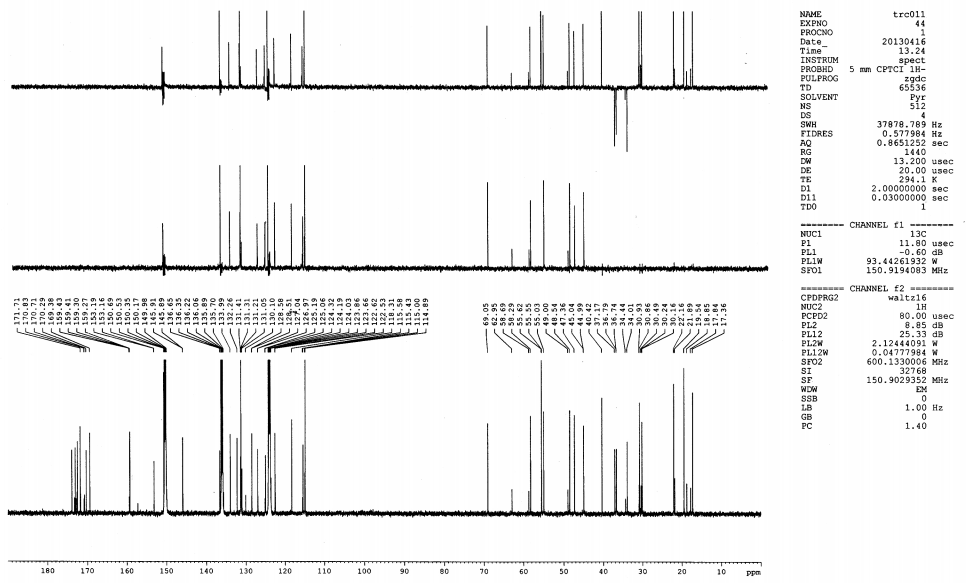


Figure N. ^13^C NMR spectra comparison of Rubipodanin A (**1,** top) and RA-V (**4,** bottom)


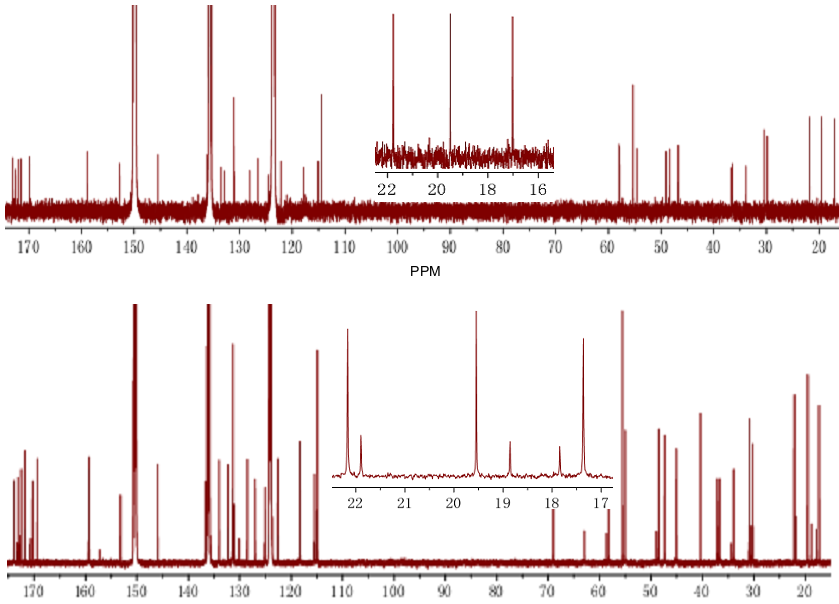

Supplement: S1 File — 1H NMR Spectrum of Rubipodanin A (1) (Figure A). 13C NMR Spectrum of Rubipodanin A (1) (Figure B). HSQC Spectrum of Rubipodanin A (1) (Figure C). COSY Spectrum of Rubipodanin A (1) (Figure D). HMBC Spectrum of Rubipodanin A (1) (Figure E). ROESY Spectrum of Rubipodanin A (1) (Figure F). ESI Mass Spectrum of Rubipodanin A (1) (Figure G). High Resolution Mass Spectrum of Rubipodanin A (1) (Figure H). UV Spectrum of Rubipodanin A (1) (Figure I). IR Spectrum of Rubipodanin A (1) (Figure J). [α]D Spectrum of Rubipodanin A (1) (Figure K). 1H NMR Spectrum of RA-V (4) (Figure L). 13C NMR Spectrum of RA-V (4) (Figure M). 13C NMR spectra comparison of Rubipodanin A (1, top) and RA-V (4, bottom) (Figure N). (DOC) [file pone.0144950.s001.doc]
